# Risk factors and drug discovery for cognitive impairment in type 2 diabetes mellitus using artificial intelligence interpretation and graph neural networks

**DOI:** 10.3389/fendo.2023.1213711

**Published:** 2023-08-25

**Authors:** Xin Zhang, Jiajia Xie, Xiong You, Houwu Gong

**Affiliations:** ^1^ Department of Pediatric, The First Affiliated Hospital of Hunan College of Traditional Chinese Medicine, Zhuzhou, China; ^2^ Department of Ultrasound Imaging, The First Hospital of Hunan University of Chinese Medicine, Changsha, China; ^3^ Center of Rehabilitation diagnosis and Treatment, Hunan Provincial Rehabilitation Hospital, Changsha, China; ^4^ College of Computer Science and Electronic Engineering, Hunan University, Changsha, China; ^5^ Military Medical Research Institute, Academy of Military Sciences, Beijing, China

**Keywords:** type 2 diabetes mellitus, cognitive impairment, risk factors, drug discovery, graph neural network (GNN)

## Abstract

**Background:**

Among the 382 million diabetic patients worldwide, approximately 30% experience neuropathy, and one-fifth of these patients eventually develop diabetes cognitive impairment (CI). However, the mechanism underlying diabetes CI remains unknown, and early diagnostic methods or effective treatments are currently not available.

**Objective:**

This study aimed to explore the risk factors for CI in patients with type 2 diabetes mellitus (T2DM), screen potential therapeutic drugs for T2DM-CI, and provide evidence for preventing and treating T2DM-CI.

**Methods:**

This study focused on the T2DM population admitted to the First Affiliated Hospital of Hunan College of Traditional Chinese Medicine and the First Affiliated Hospital of Hunan University of Chinese Medicine. Sociodemographic data and clinical objective indicators of T2DM patients admitted from January 2018 to December 2022 were collected. Based on the Montreal Cognitive Assessment (MoCA) Scale scores, 719 patients were categorized into two groups, the T2DM-CI group with CI and the T2DM-N group with normal cognition. The survey content included demographic characteristics, laboratory serological indicators, complications, and medication information. Six machine learning algorithms were used to analyze the risk factors of T2DM-CI, and the Shapley method was used to enhance model interpretability. Furthermore, we developed a graph neural network (GNN) model to identify potential drugs associated with T2DM-CI.

**Results:**

Our results showed that the T2DM-CI risk prediction model based on Catboost exhibited superior performance with an area under the receiver operating characteristic curve (AUC) of 0.95 (specificity of 93.17% and sensitivity of 78.58%). Diabetes duration, age, education level, aspartate aminotransferase (AST), drinking, and intestinal flora were identified as risk factors for T2DM-CI. The top 10 potential drugs related to T2DM-CI, including Metformin, Liraglutide, and Lixisenatide, were selected by the GNN model. Some herbs, such as licorice and cuscutae semen, were also included. Finally, we discovered the mechanism of herbal medicine interventions in gut microbiota.

**Conclusion:**

The method based on Interpreting AI and GNN can identify the risk factors and potential drugs associated with T2DM-CI.

## Introduction

Cognition is the natural process whereby the brain recognizes and acquires information (1). Cognitive impairment (CI) refers to decreased cognitive processing speed and efficiency, affecting functions such as working memory, task execution, and attention (2). Memory impairment is the most common cognitive change and may progress to dementia in severe cases (3). In recent years, CI has become increasingly recognized as one of the most important cerebrovascular complications of type 2 diabetes (T2DM) (4). There is an increasing consensus suggesting that T2DM is one of the most important causes of CI (5), with reports suggesting that diabetes can lead to a 20%–70% decline in cognitive ability, and the risk of dementia is 60% higher in diabetic patients than in non-diabetic patients (6). Diabetes is the most prevalent metabolic disease worldwide, with 500 million T2DM patients globally, one-third of whom are in China (7). With the changing social structure and the global aging trend, the number of CI cases caused by T2DM is expected to increase exponentially. Studies have shown that the incidence of mild CI in T2DM patients is significantly higher than in non-diabetic patients (8, 9). Mild CI may affect daily activities, such as impaired intelligence, slow thinking speed, reduced flexibility, and lack of concentration (10). CI caused by diabetes can be classified into diabetes-related cognitive decline, mild CI (MCI), and dementia according to severity (11). Therefore, CI can be considered as an intermediate transition between diabetes and dementia, and this process is reversible. Therefore, it is urgent to identify the risk factors for T2DM-CI and prevent its occurrence and development. Research on the risk factors for T2DM-CI has gained significant momentum in recent years. However, no consensus has been reached, and the literature has been predominantly based on foreign populations. The risk factors for T2DM-CI in China have been largely underinvestigated, and the clinical and demographic data included are not comprehensive and cannot reflect the real risk factors for T2DM patients with CI. This study aims to comprehensively analyze the risk factors for T2DM-CI, focusing on demographic characteristics and relevant clinical and physical indicators, to identify T2DM patients with possible CI early, discover potential drugs, improve patient quality of life, and reduce the burden on society.

## Materials and methods

### Study design and patients selection

The study included a population of patients with type 2 diabetes mellitus (T2DM) who were admitted to the Endocrinology Department of the First Affiliated Hospital of Hunan College of Traditional Chinese Medicine and the First Affiliated Hospital of Hunan University of Chinese Medicine between January 2018 and December 2022, and who met the specified inclusion criteria. The selection of research subjects involved a rigorous screening process conducted by at least two medical professionals, who assessed the patients using cognitive scales. Based on the assessment criteria, the patients were divided into two groups: the T2DM group with normal cognition (T2DM-N group) and the T2DM group with cognitive impairment (T2DM-CI group).

### Diagnostic criteria

The diagnosis criteria for T2DM were based on the “Chinese Guidelines for the Prevention and Treatment of Type 2 Diabetes (2013 edition)” (12). According to these criteria, T2DM can be diagnosed if patients presenting with diabetes-related symptoms (such as polyphagia, polydipsia, polyuria, and unexplained weight loss) meet any of the following three conditions: (1) random blood glucose (blood glucose at any time within a day) ≥11.1mmol/L; (2) fasting blood glucose (without calorie intake in 8 h) ≥ 7.0mmol/L; (3) blood glucose value ≥ 11.1mmol/L measured 2 h after 75 g oral glucose tolerance test. For individuals without diabetes symptoms, the blood glucose is re-tested on another day to confirm the diagnosis.

The diagnostic criteria for cognitive impairment are based on the 5th edition of the “Diagnostic and Statistical Manual of Mental Disorders” (DSM-5) and the official manual of the Montreal Cognitive Assessment (MoCA) scale (13). The following three conditions must be met to diagnose cognitive impairment: (1) The Chinese version of the MoCA score is<26 points; (2) the patient, their family, or those who know the patient well provide relevant descriptions of memory decline; (3) the patient has basic daily living abilities, with a score ≥16 on the instrumental activities of daily living scale (IADL).

### Inclusion and exclusion criteria

The inclusion criteria for the study population were as follows:

1. T2DM-N group: patients diagnosed with type 2 diabetes mellitus according to the diagnostic criteria outlined in the “China Type 2 Diabetes Prevention and Control Guidelines (2013 Edition).”
2. T2DM-CI group: patients diagnosed with both T2DM and cognitive impairment.
3. Age between 30 and 85 years.
4. T2DM disease duration of more than 1 year.
5. Patients with complete data on relevant indicators.

The exclusion criteria were as follows:

1. Patients who have experienced acute metabolic complications of diabetes, such as diabetic ketoacidosis or hyperosmolar hyperglycemic state, within the past month.
2. Patients who recently experienced diseases that may affect glucose and lipid metabolism, such as infection, trauma, stress, or surgery.
3. Patients with severe cardiovascular disease, hematological system disease, malignant tumor, or other serious primary diseases, severe liver or kidney dysfunction, or mental illness.
4. Patients who have experienced serious acute brain diseases within the past 3 months, such as acute cerebral infarction, intracranial hemorrhage, or acute meningitis.
5. Pregnant or lactating women, or those planning to become pregnant.
6. Patients who have participated in other clinical trials within the past 3 months.

### Analysis variables

This study extracted patients’ personal information and laboratory examination data from the hospital information system. The analyzed variables included gender, age, body mass index (BMI), heart rate, blood pressure, duration of type 2 diabetes mellitus (T2DM), family history, smoking and drinking history, exercise habits, and more. Laboratory indicators encompassed total cholesterol (TC), triglycerides (TGs), high-density lipoprotein cholesterol (HDL-C), low-density lipoprotein cholesterol (LDL-C), very-low-density lipoprotein cholesterol (VLDL-C), homocysteine (HCY), fasting blood glucose (FBG), 2-h postprandial blood glucose (2hPBG), glycosylated hemoglobin (HbA1c), fasting plasma insulin (FINS), fasting C-peptide, creatinine (Crea), aspartate aminotransferase (AST), alanine aminotransferase (ALT), and gut microbiota. In addition, this study utilized data from various databases, including Traditional Chinese Medicine Systems Pharmacology (TCMSP) (14), Online Mendelian Inheritance in Man (OMIM) (15), Therapeutic Target Database (TTD) (16), Pharmacogenomics Knowledgebase (Pharm Gkb) (17), and Drug Bank (18), to conduct drug discovery research for T2DM-CI.

### Machine learning methods

The raw data were processed by organizing and standardizing them. Any feature with missing values exceeding 50% was removed from the dataset. For the remaining features with missing values, continuous features were imputed using the mean and categorical features using the mode. Six machine learning models were selected as candidates for analysis, which included random forest (RF), gradient boosted decision tree model (GBDT), light gradient boosting machine (LGBM), extreme gradient boosting (XGBoost), and categorical features gradient boosting (CatBoost) (19).

- Random Forest is an algorithm that utilizes multiple decision trees to train and predict samples. The output category is determined by the mode of the individual decision tree output categories. Random Forest is insensitive to missing values, capable of handling imbalanced data, and exhibits robustness to outliers.
- Gradient Boosting Decision Tree (GBDT) is a boosting ensemble algorithm based on decision trees incorporating gradient descent. The algorithm consists of multiple decision trees, and the conclusions of all trees are accumulated to provide the final answer. GBDT can handle various types of data, including continuous and discrete values, in a flexible manner. It exhibits high prediction accuracy with relatively less parameter tuning time. Moreover, it demonstrates strong robustness to outliers by utilizing robust loss functions.
- LightGBM is a decision tree algorithm based on histograms, which transforms the storage of feature values into the storage of bin values and does not require the indexing of feature values to samples. LightGBM employs an exclusive feature bundling algorithm to reduce the number of features during the training process, resulting in exceptionally fast training speeds. Therefore, it is highly suitable for classification problems involving high-dimensional datasets.
- XGBoost is a boosting algorithm based on CART trees. XGBoost uses the second-order Taylor expansion of the loss function as a surrogate function, which is then minimized to determine the optimal split point and leaf node output value of the regression tree. XGBoost offers reduced learning time and exhibits high flexibility in its approach.
- CatBoost is an algorithm that utilizes symmetric decision trees (oblivious trees) as its base learner. It incorporates a specialized method to handle categorical features and employs ordered boosting with combined categorical features to prevent gradient estimation bias. CatBoost demonstrates exceptional performance, reduces the need for hyperparameter tuning, and exhibits strong robustness.

The characteristics of logistic regression are simple calculation and strong interpretability, which are widely used in fields such as finance, healthcare, social networks, and marketing. Random Forest is characterized by no need for feature normalization and feature selection. Random Forest is mainly used for training sets with high square error and low deviation. The characteristics of Adaboost are low generalization error rate, easy coding, and sensitivity to outliers. Adaboost is suitable for baseline classification tasks. CatBoost is particularly adept at handling category features. CatBoost is suitable for processing categorical data. The characteristic of GBDT is high prediction accuracy, suitability for low dimensional data, and ability to handle nonlinear data. GBDT is applicable to regression problems (linear and nonlinear), and it is also applicable to binary classification problems and multiclassification problems. The characteristic of XGBoost is its support for parallel computing, fast training speed, suitability for high bias, low variance training sets, and suitability for numerical vectors.

The entire dataset was randomly split into an 80% training set and a 20% testing set for model training and evaluation. Performance metrics from the validation set were utilized to compare the models and estimate their generalization ability. The Shapley method was employed to enhance the interpretability of the model, providing insights into the factors influencing T2DM-CI at a local level. Furthermore, a graph neural network model was utilized for drug discovery research on T2DM-CI, identifying potential therapeutic drugs with beneficial effects on T2DM-CI.

### Evaluation indicators

This study employed k-fold cross-validation for model validation to evaluate the robustness of the models. The training set was divided into K subsets, with one subset reserved as the validation data, while the remaining K-1 subsets were used for model training. The cross-validation process was repeated K times, with each subset being used as the validation set once, and the results were averaged or combined using other methods to obtain a single estimate. The key advantage of this method is that it repeatedly utilizes randomly generated subsets for training and validation, ensuring a comprehensive evaluation of the models. In this study, the value of k was set to 5.

The experiment adopts the area under the ROC curve (AUC) as the main evaluation indicator and specificity (Spe) and sensitivity (Sen) as secondary indicators. The higher the specificity, the higher the probability of accurate diagnosis; the higher the sensitivity, the lower the probability of missed diagnosis. The calculation formula is as follows:


1 $$Spe=\frac{TN}{FP+TN}$$

2 $$Sen=\frac{TP}{TP+FN}$$

where TP represents the number of true positive samples, TN represents the number of true negative samples, FP represents the number of false-positive samples, and FN represents the number of false-negative samples.

### Statistical analysis

The statistical analysis in this study was conducted using SPSS 22.0 software. Continuous data were reported as mean ± standard deviation ( $\bar{x}\pm s$ ). Prior to analysis, normal distribution and homogeneity of variance tests were performed. If the data satisfied the assumptions of normal distribution and homogeneity of variance, t-tests or ANOVA were employed for analysis. On the other hand, if the data did not meet these assumptions, non-parametric Wilcoxon rank sum tests were utilized. The comparison of count data was assessed using a chi-square test. A p-value< 0.05 was statistically significant.

### GCNN4Micro-Dis model for discovery of potential drugs

We obtained 269 drugs, 598 diseases, and 18,416 disease–drug associations from the Comparative Toxicology Database (CTD). Then, we obtained more information from LTM-TCM, including 1,928 disease symptoms, 9,122 herb medicines, and 1,170,133 associations. In this study, the performance parameters of the ROC and AUPR curves are used as the criteria for selecting drugs based on the graph neural network model. The GCNN4Micro-Dis model evidently performed well and can help identify potential disease–drug associations. The correlation scores were calculated through the model to ensure the relevance between the selected drugs and T2DM-CI.

**Figure 1:**
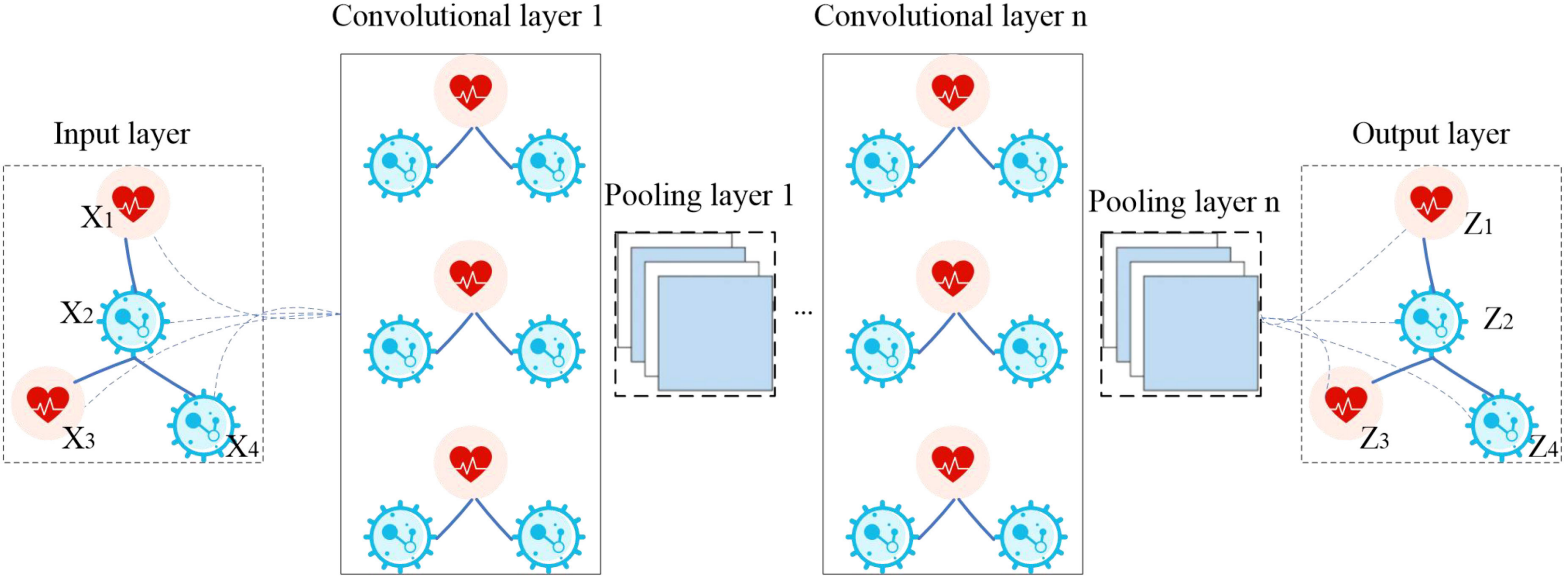
The flowchart of GCNN4Micro-Dis.

The model GCNN4Micro-Dis (20), previously developed by a research team, was used to predict potential drugs. The structure of GCNN4Micro-Dis is shown in **Figure 1**. The model consists of three main steps: (1) performing a graphic Fourier transform on the input data, (2) convolving the transformed result in the spectral domain, and (3) processing the convolution result using inverse Fourier transform.

## Results

### Demographic and clinical characteristics of study participants

This study involved 719 patients, with 255 (33.62%) diagnosed with type 2 diabetes cognitive impairment and 464 (66.38%) without cognitive impairment. A comparison of the data between patients with and without the endpoint event indicated no significant differences in gender, BMI, smoking, total cholesterol (TC), triglycerides (TGs), and other variables (p>0.05). However, significant differences were observed in age, education level, duration of diabetes, hypertension, intestinal flora, and LDL-C value (p<0.05). More details are provided in **Table 1**.

**Table 1:** Comparison of information among T2DM patients.

	T2DM-CI (n=255)	T2DM-N (n=464)	χ^2^/t/Z	p-value
Gender			1.34	0.26
Male	135(52.94%)	244(52.59%)		
Female	120(47.06%)	220(47.41%)		
Age (year)	64.32±8.32	60.12±10.85	−3.37	<0.01
Education level			18.51	<0.01
Below middle	110(43.14%)	166(35.78%)		
Middle and above	145(56.86%)	298(64.22%)		
BMI(kg/m^2^)	24.01±2.25	25.93±3.12	2.53	0.08
Diabetes duration (year)	13.85±8.11	11.36±6.49	−0.37	<0.01
Smoke			0.02	0.96
No	131(51.37%)	221(47.63%)		
Yes	124(48.63%)	243(52.37%)		
Drink			1.12	0.04
No	117(45.88%)	207(44.61%)		
Yes	138(54.12%)	257(55.39%)		
Hypertension			7.29	<0.01
Normal	129(50.59%)	231(49.78%)		
Abnormal	126(49.41%)	233(50.22%)		
Cerebral infarction			8.28	<0.01
Normal	117(45.88%)	196(42.24%)		
Abnormal	138(54.12%)	268(57.76%)		
Intestinal flora			7.67	<0.01
Normal	129(50.59%)	188(40.52%)		
Abnormal	126(49.41%)	276(59.48%)		
TC (mmol/L)	8.91±3.01	8.85±3.65	−0.53	0.61
TG (mmol/L)	11.79±3.04	11.37±3.86	−0.96	0.38
LDL-C (mmol/L)	11.15±9.93	16.21±10.98	−1.88	0.04
HCY (µmol/L)	13.72±10.41	14.73±11.28	−0.83	0.45
FBG (mmol/L)	9.11±3.44	9.01±3.81	−0.57	0.59
2hPBG (mmol/L)	11.85±3.53	11.48±3.77	−0.89	0.34
HbA1c (%)	9.44±2.04	9.13±1.99	−1.89	0.05
FINS (µIU/ml)	12.55±10.21	16.94±11.17	−1.76	0.03
HoMA-IR	4.64±4.11	7.94±4.73	−0.37	0.74
Crea (µmol/L)	73.83±27.36	72.11±21.66	−0.55	0.63
AST (U/L)	15.75±8.23	18.93±13.84	−1.84	0.04
ALT (U/L)	15.42±7.79	17.91±7.26	−0.72	0.47

### Comparison of performance of T2DM-CI risk prediction models

**Figure 2:**
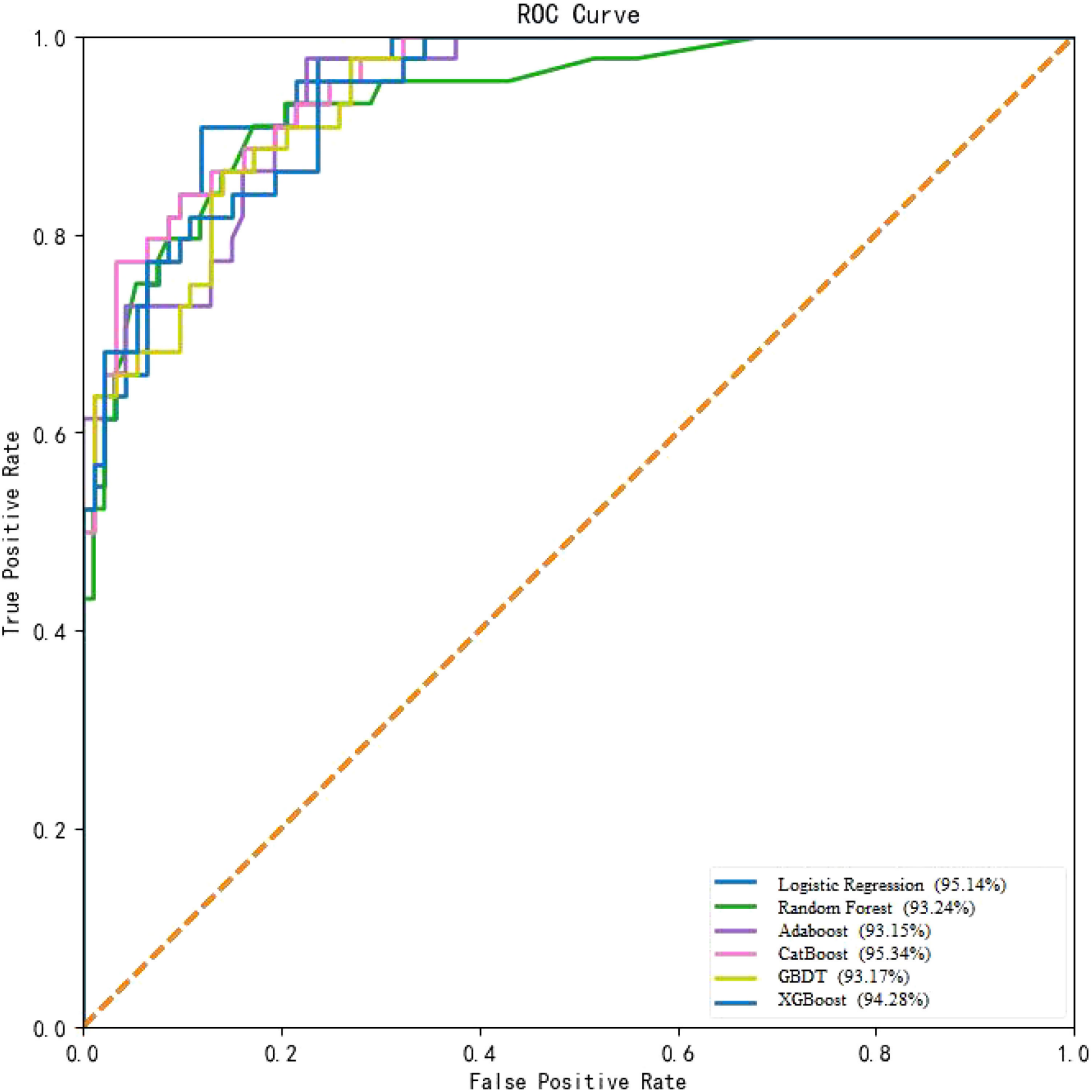
ROC curves of different machine learning algorithms on the test set.

In this study, the performance of six machine learning algorithms, namely, Logistic Regression, Random Forest, GBDT, Adaboost, XGBoost, and CatBoost, was compared in predicting the risk of T2DM-CI. The results (**Table 2**, **Figure 2**) showed that CatBoost exhibited higher AUC and Spe values than the other models in the validation set. The AUC value in the validation set was 95.34%, surpassing the AUC values of the other five models. Additionally, the specificity was 93.17%, outperforming the other four models. The Random Forest model achieved the highest sensitivity (78.58%). Overall, the experimental data from this study demonstrated that the CatBoost model was superior to other models in predicting the risk of T2DM-CI.

**Table 2:** Comparison of results among different machine learning algorithms.

DataSet	Algorithms	AUC (%)	Specificity(%)	Sensitivity(%)
Training set	Logistic regression	96.94	93.76	80.35
	Random Forest	99.99	99.99	99.99
	GBDT	99.46	98.83	90.32
	Adaboost	98.35	96.18	88.86
	XGBoost	99.99	99.99	99.99
	CatBoost	99.81	98.12	94.51
Test set	Logistic regression	95.14	90.92	77.27
	Random Forest	93.24	91.27	**78.58**
	GBDT	93.17	90.23	72.73
	Adaboost	93.15	91.15	72.73
	XGBoost	94.28	91.19	77.27
	**CatBoost**	**95.34**	**93.17**	77.27

The bold values means the highest value.

### Discovery of risk factors for T2DM-CI

**Figure 3:**
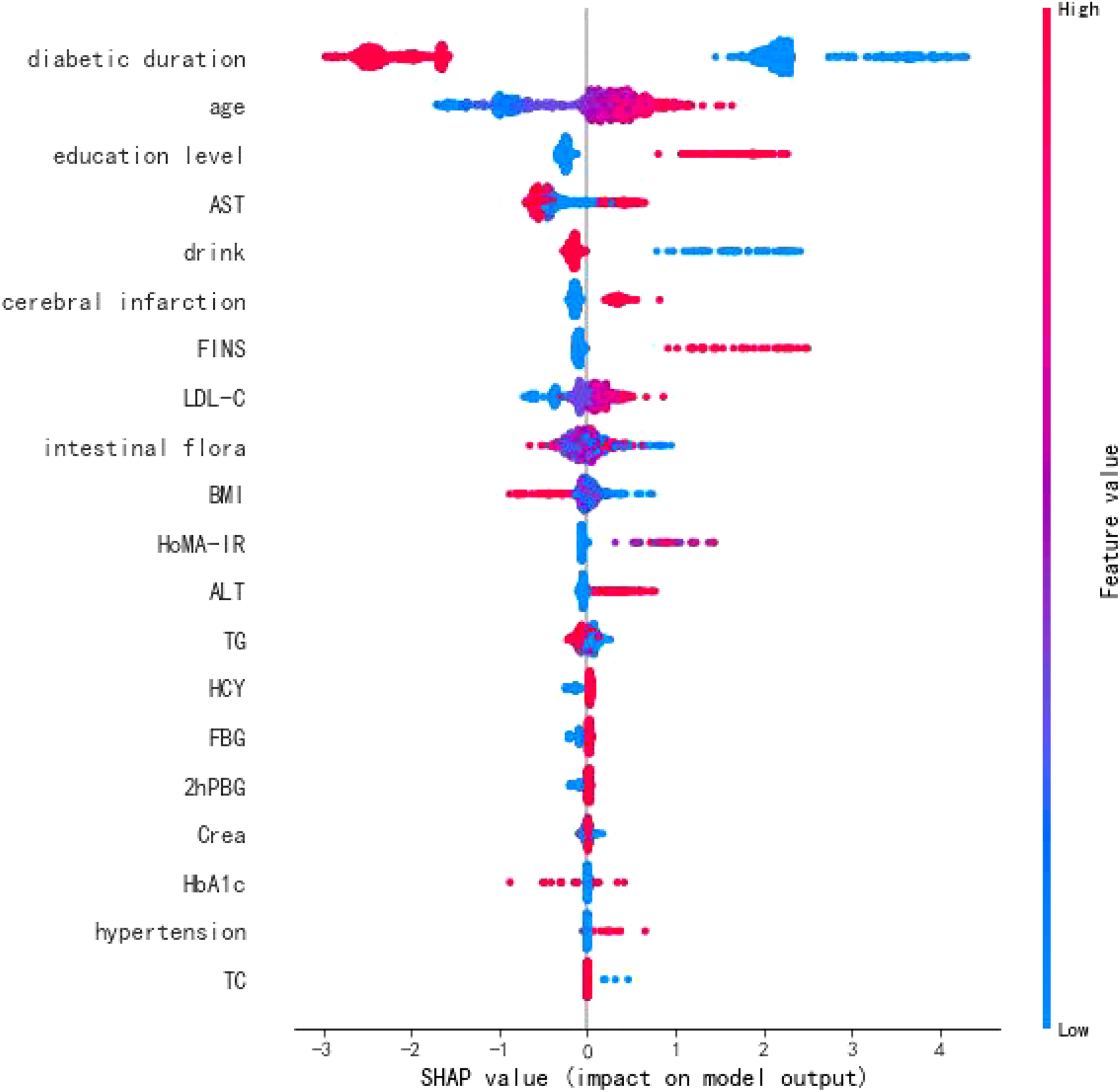
T2DM-CI features importance.

To explore the risk factors influencing T2DM-CI, this study introduced an interpretive T2DM-CI prediction model based on CatBoost and TreeSHAP (21). From a global perspective, the importance of features contributing to T2DM-CI was ranked and presented in **Figure 3**. The analysis revealed that T2DM-CI might be associated with factors such as diabetes duration, age, education level, AST, drinking habits, and intestinal flora.

### Discovery of potential drugs related to T2DM-CI

In the previous section, intestinal flora was identified as a risk factor for T2DM-CI. In this section, we analyzed the relationship between “T2DM-CI_intestinal flora_drug.” Subsequently, we utilized the GCNN4Micro-Dis model (20) to identify potential drugs associated with T2DM-CI. **Table 3** presents the top 10 drugs ranked by their association scores with T2DM-CI. Some herbs were included, such as licorice and cuscutae semen. It is worth mentioning that the results obtained have been validated in the published literature (22).

**Table 3:** Top 10 potential drugs related to T2DM-CI.

Rank	Related drugs	Scores	Evidence
1	Metformin	0.00061	PMID: 31975558
2	Liraglutide	0.00060	PMID: 31790314
3	Lixisenatide	0.00059	PMID: 21391833
4	Liquorice	0.00058	PMID: 36232291
5	Dulaglutide	0.00057	PMID: 30394576
6	3-n-Butylphthalide	0.00056	Unconfirmed
7	Cuscutae Semen	0.00056	Unconfirmed
8	Lycii Fructus	0.00055	PMID: 16689001
9	DPP-4i	0.00054	PMID: 30394576
10	Rhizoma Dioscoreae	0.00054	PMID: 31717456

## Discussion

In this study, our approach based on artificial intelligence interpretation and graph neural networks enabled the identification of risk factors and potential drugs that impact the progression of T2DM to cognitive impairment. These findings offer valuable insights for the comprehensive treatment of T2DM and the prevention of dementia. The analysis highlighted the significance of diabetes duration, age, education level, AST, alcohol consumption, and intestinal flora as important risk factors for T2DM-CI. Importantly, the present study focused on the T2DM population and assessed relevant risk factors, enabling more accurate and convenient screening and early prevention in clinical practice. Furthermore, this study encompassed a comprehensive range of potential risk indicators. While previous research primarily concentrated on common clinical indicators, this study incorporated emerging potential risk indicators such as HoMA-IR, FINS, and intestinal flora. This expansion of the risk screening scope provides a valuable reference value for future research and enhances our understanding of the multifaceted nature of T2DM-CI.

However, it should be borne in mind that this study has some limitations. The available case data were limited, which restricted the ability to conduct a stratified analysis of certain influencing factors, and the findings may be biased to some extent. Therefore, our results can only reflect the influencing factors of cognitive impairment in the T2DM population to some extent and should be interpreted with caution. Nonetheless, the findings still provide valuable guidance for preventing and treating cognitive impairment in T2DM patients. Clinical data comprise patient visit information, yet accurately reflecting all patients’ symptoms through electronic medical records can be challenging for doctors, resulting in incomplete data. Indeed, some symptoms that go unnoticed by doctors may go unrecorded, leading to missing records in hospital documentation of patient visits. Furthermore, different hospitals may have varying records for the same disease, and symptoms can vary among patients. Consequently, there is a limited availability of clinical samples for real-world data. The sample size in this study was determined based on the existing data, without prior power calculation for sample size. Consequently, the study is limited by a small sample size of clinical samples, which impacts the research quality. To enhance the robustness of the results, this study necessitates a larger sample size and a more standardized research paradigm. On the one hand, we plan to explore alternative methods to increase the sample size or utilize additional data sources from public databases to complete multicenter validation studies, such as the Pima Indians Diabetes Database. On the other hand, we plan to create a questionnaire and distribute it to third-party survey teams, such as the PowerCX Wind Chime System, which can target a sample of people to answer the questionnaire. Over the past decade, third-party survey teams have become increasingly popular and even trusted by professional research companies. With the advent of big data and the continuous improvement of multisystem network connections, favorable conditions should be established to facilitate further research into the influencing factors. This will contribute to the generation of more optimized clinical evidence, enabling a deeper understanding of the complex interactions and variables involved in various medical conditions.

The results of this study highlight several important findings regarding the relationship between type 2 diabetes and mild cognitive impairment. First, the duration of diabetes was identified as a potential risk factor for cognitive impairment. A longer duration of diabetes (more than 20 years) was associated with a higher likelihood of cerebral vascular injury, brain atrophy, and impaired cognitive function. This can be attributed to the chronic metabolic dysfunction associated with diabetes, which leads to ischemic and hypoxic changes in brain tissue and increased inhibitory neurotransmitters (23). Additionally, age was a significant factor in the development of mild cognitive impairment in patients with type 2 diabetes. Older patients, particularly those between 60 and 75, were more susceptible to cognitive impairment. This observation is consistent with previous research, suggesting that age-related decline in dopamine neurotransmission efficiency and frontal gyrus system function contribute to the deterioration of cognitive function over time (24).

Furthermore, education level was identified as a strong determinant of cognitive impairment in individuals with type 2 diabetes. Higher education levels were associated with better cognitive function, attributed to engaging in intellectual labor, maintaining good learning habits, and keeping brain cells active. Conversely, lower education levels, often associated with more physical labor and limited brain usage, led to a decline in brain neuron reserve and decreased awareness of health management (25).

**Figure 4:**
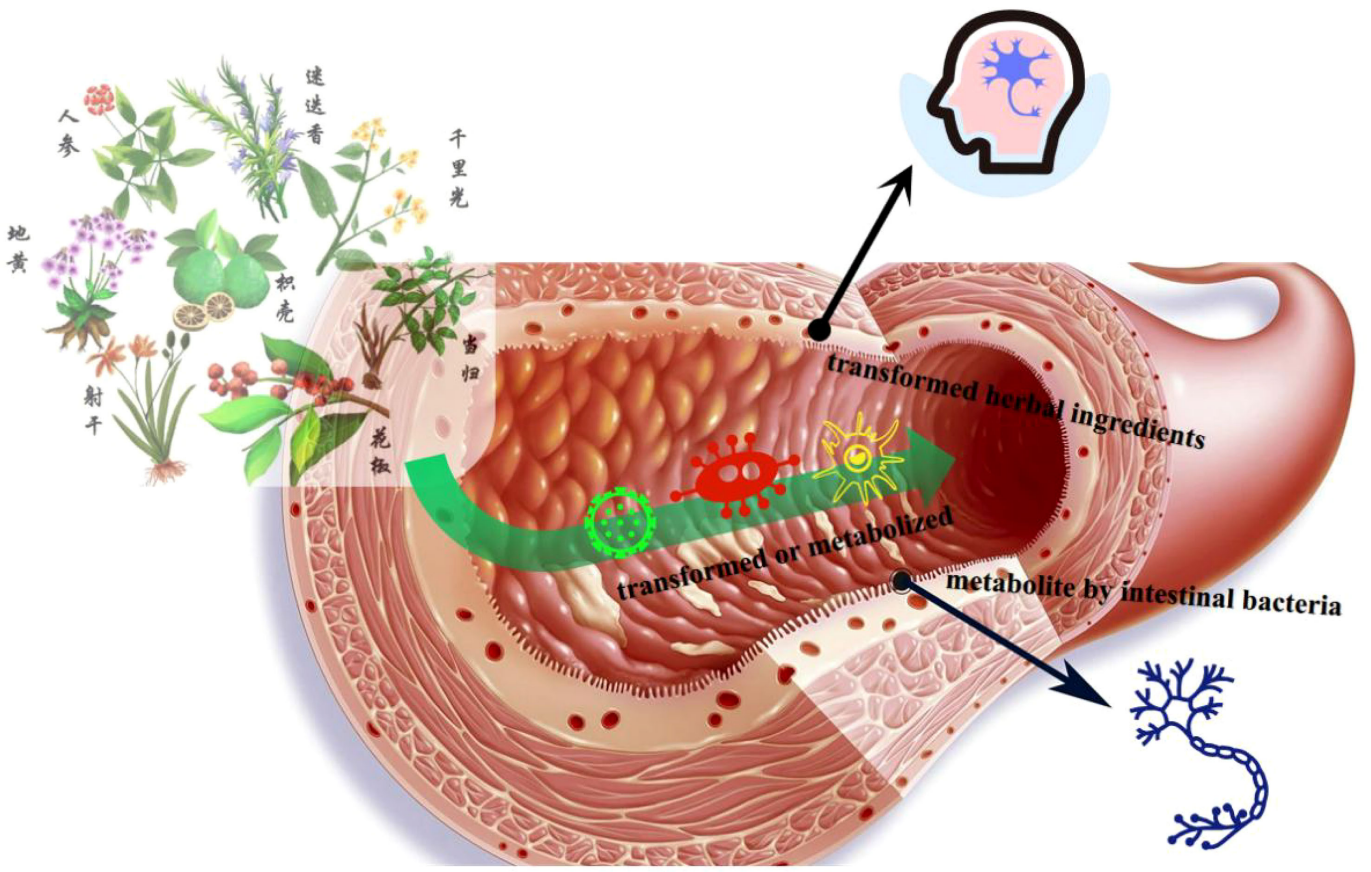
Mechanism of herbal medicine interventions in gut microbiota.

Furthermore, this study revealed that intestinal flora may be a potential risk factor for mild cognitive impairment in patients with type 2 diabetes. Intestinal flora primarily influences the host through its bacterial bodies and metabolic byproducts (26). Intestinal dysbiosis in individuals with diabetes can directly affect central function and promote other pathways that impact cognitive function. These pathways are interconnected. Intestinal flora can influence metabolic and neurological diseases, offering a novel perspective for treating T2DM-CI. The altered flora in diabetic patients plays a crucial role in their cognitive impairment, highlighting the potential of regulating intestinal flora as an effective treatment target for T2DM-CI (**Figure 4**).

Most traditional Chinese medicine formulas can modulate the composition of the symbiotic flora. A multicenter, randomized, open-label clinical trial demonstrated that a combination of metformin and a traditional Chinese medicine formula containing *Salvia miltiorrhiza*, *Anemarrhena asphodeloides*, *Schisandra chinensis*, *Coptis chinensis*, red yeast rice, aloe vera, bitter melon, and dried ginger could improve type 2 diabetes with hyperlipidemia by promoting the growth of beneficial flora, such as Blautia and Faecalibacterium (27). Furthermore, another Chinese medicine formula Ge-Gen-Qin-Lian decoction, has been found to enrich beneficial flora, including Faecalibacterium, in the gut, associated with its anti-diabetic effects (28). Chinese medicine exerts its regulatory effects through intricate chemical interactions in the gut, thereby maintaining a healthy gut ecosystem, controlling insulin resistance, and reducing host inflammation.

Considering further experimental validation of our results, the planned experiments and validation methods are as follows. First are the molecular and cellular experiments. *In vitro* experiments involve applying this candidate drug to the cell model of the relevant disease, observing whether it can affect the related pathological changes of this disease model. The techniques we may use include immunofluorescence staining, Western blot, qPCR, etc., to detect changes in key biomarkers. Second are animal experiments. If *in vitro* experiments prove that the drug has an effect on specific targets or pathways, then *in vivo* research is conducted, usually in animal models. At this stage, we need to observe whether the administration of the candidate drug in a specific disease model can improve symptoms or pathological changes. Third are clinical trials. If in both *in vitro* and *in vivo* experiments, the drug demonstrates the potential to alter biological processes and exhibits good safety, a clinical trial is then conducted to verify the drug’s effects and safety in humans. This is a key step in our final confirmation of the drug’s applicability and safety.

## Data availability statement

The original contributions presented in the study are included in the article/supplementary material. Further inquiries can be directed to the corresponding author.

## Author contributions

Study concept and design: HG. Acquisition of data: XZ, JX. Analysis and interpretation of data: XY. Drafting of the manuscript: XZ, JX. Critical revision of the manuscript for important intellectual content: HG. Statistical analysis: XY. Obtained funding: HG. Technical or material support: XZ, JX. Study supervision: HG. All authors contributed to the article and approved the submitted version.
